# Exploiting the predictive power of educated spheroids to detect immune-mediated idiosyncratic drug-induced liver injury: the case of troglitazone

**DOI:** 10.3389/fphar.2024.1378371

**Published:** 2024-04-10

**Authors:** Salomé Roux, Sara Cherradi, Hong Tuan Duong

**Affiliations:** PredictCan Biotechnologies SAS, Biopôle Euromédecine, Grabels, France

**Keywords:** educated spheroid model, drug-induced liver injury, idiosyncratic, immune-mediated, idiosyncratic drug-induced liver injury, risk prediction, high-throughput screening, companion test

## Abstract

Idiosyncratic drug-induced liver injury (iDILI) is a major concern in drug development because its occurrence is unpredictable. Presently, iDILI prediction is a challenge, and cell toxicity is observed only at concentrations that are much higher than the therapeutic doses in preclinical models. Applying a proprietary cell educating technology, we developed a person-dependent spheroid system that contains autologous educated immune cells that can detect iDILI risk at therapeutic concentrations. Integrating this system into a high-throughput screening platform will help pharmaceutical companies accurately detect the iDILI risk of new molecules de-risking drug development.

## 1 Introduction

Drug-induced liver injury (DILI) is a main cause of drug development failures and market withdrawals. Despite the availability of a large panel of *in vitro* and animal models to screen for DILI risk, its detection remains problematic as it does not occur in each person. Indeed, idiosyncratic DILI (iDILI) is usually dose-independent and occurs in only a small fraction of subjects. Furthermore, there is evidence that most iDILI cases are immune-mediated, suggesting that its occurrence may also depend on the age and sex of the subject as these non-genetic factors influence the immune cell landscape (Uetrecht, 2019; Huang et al., 2021; Allison et al., 2023).

Troglitazone (TGZ) is an antidiabetic drug that was removed from the market because of severe cases of liver failure. During its development, no hepatotoxicity was detected. Nevertheless, once on the market, severe cases of liver injury leading to liver transplant or death were observed (Funk et al., 2001; Chojkier, 2005). This evidence suggests that iDILI is a difficult-to-detect event at early stages of drug development presumably due to the poor predictivity of available models. Indeed, existing *in vitro* models, including primary human hepatocytes (PHHs), iPSC-derived liver organoids, and animal models, failed to predict the potential of TGZ to induce severe liver injury in a larger patient population (Segovia-Zafra et al., 2021; Stern et al., 2023). The mechanism of liver injury caused by troglitazone remains unclear. However, data show that troglitazone upregulates cytochrome P450 CYP3A4 expression and is metabolized into reactive metabolites that cause hepatocyte damage and release of damage-associated molecular patterns (DAMPs), which then trigger macrophage inflammasome activation and liver injury (Sahi et al., 2000; Kato and Uetrecht, 2017; Mak et al., 2018). These results suggest that TGZ is an immune-mediated DILI compound. TGZ-mediated iDILI was not predicted accurately during drug discovery. Consequently, TGZ was removed from the market 3 years after its approval due to liver failure. It is, therefore, obvious that the lack of a good predictive model for iDILI risk constitutes a bottleneck for drug development.

We recently developed a subject-dependent educated spheroid system that can detect the DILI risk of difficult-to-detect molecules (Cherradi et al., 2023). Unlike the cell reprogramming technique, our cell educating technology does not modify the cell fate but induces a specific behavior in the cells owing to the composition of the serum supplemented in the cell culture. These cells are called educated cells throughout the study. In the present work, we used this technology to add educated autologous monocyte-derived macrophages and dendritic cells (DCs) into our hepatic spheroids and demonstrated that our system can detect TGZ-mediated iDILI at therapeutic doses. Moreover, we confirmed that our system can detect immune-mediated iDILI of diclofenac. Our easy-to-handle and quick-to-set-up educated spheroid system opens perspectives for high-throughput screening (HTS) of the iDILI risk of new compounds and for its use as a companion test to identify patients at risk for iDILI prior to the initiation of the treatment protocol.

## 2 Materials and methods

### 2.1 Biological samples, cell lines, and reagents

Patients’ blood was collected and processed by mechanical filtration according to the protocol described elsewhere (Cherradi et al., 2023). Blood samples from healthy donors (>18 years, >50 kg body weight, no viral infection, no chronic disease, and no cancer) were obtained from the Etablissement Français du Sang (EFS) Hauts de France, Normandy. Blood samples from 96 healthy individuals were used in this study. A total of 72 samples were used to generate educated spheroids to assess the iDILI risk of troglitazone and its non-iDILI partner rosiglitazone (RGZ), and 24 samples were used to prepare spheroids for diclofenac and acetylsalicylic acid analysis.

The research protocol was conducted under French legal guidelines and fulfilled the requirements of the local institutional ethics committee. The study was approved by the “Direction Générale de la recherche et de l’innovation” (CODECOH, n°DC-2021-4779). This project does not involve the human subjects according to the legislation (article L1121-1 du code de la santé publique). TGZ, RGZ, diclofenac, and acetylsalicylic acid (ASA) were purchased from CliniSciences (Nanterre, France).

Hepatocyte (HepG2) and monocyte (THP-1) cell lines were obtained from ATCC (Molsheim, France). The hepatic stellate cell line (TWNT-1) was obtained from Glow Biologics (Tarrytown, NY, United States). Cell culture reagents were provided by STEMCELL (Saint Égrève, France). Hepatocytes, monocytes, and hepatic stellate cells were conditioned for a minimum of 2 weeks in MammoCult^®^ basal medium (STEMCELL) before use to sensitize them to the cell educating technology.

Educated monocytes were prepared for each healthy donor in OneSmartDiff medium (PredictCan Biotechnologies, Grabels, France), a customizable person-dependent medium. The OneSmartDiff medium is a medium defined on the basis of the MammoCult^®^ basal medium containing a broad-spectrum antibiotic supplemented with serum prepared from each individual. The absence of *Mycoplasma* contamination was verified using the MycoAlert^®^ *Mycoplasma* Detection Kit (Lonza, Saint-Beauzire, France).

### 2.2 Educated spheroid preparation

Educated spheroids were generated from a co-culture of HepG2 and TWNT-1 cells in ultra-low attachment plates using blood from healthy donors. The system was supplemented with educated monocytes and cultured for 3 days before being treated for 3 days with TGZ or RGZ. Importantly, educated spheroids and educated monocytes were prepared from the same donor and then co-cultured together.

### 2.3 Treatments

Educated spheroids were treated with troglitazone or rosiglitazone at concentrations up to 100× C_max_. C_max_ values were obtained from clinical pharmacokinetic studies (Loi et al., 1999; Plosker and Faulds, 1999; Chapelsky et al., 2003; Nagelschmitz et al., 2014; Chen et al., 2015; Day and Bailey, 2016). TGZ C_max_ = 6.39 µM, RGZ C_max_ = 1.04 µM, diclofenac C_max_ = 7.44 µM, and ASA C_max_ = 5.52 µM. The cell viability was then measured after 3 days of treatment. For each drug, we generated an inhibitory dose–response curve fit with constraints (top = 100; bottom = 0) and calculated the LogIC_50_ and HillSlope values.

### 2.4 Bright-field microscopy and cell counting

THP-1 cells were educated in donor-dependent OneSmartDiff medium for 3, 6, and 8 days. The cells were washed once with PBS to remove non-adherent cells. Pictures were taken of monocyte-derived macrophages and dendritic cells on different days. Cell populations were counted using ImageJ.

### 2.5 Cell viability

Cell viability was measured using CellTiter-Glo (Promega, Charbonnières-les-Bains, France) according to the manufacturer’s instructions.

### 2.6 Alanine aminotransferase quantification

Educated spheroids were cultured for 3 days and then treated for another 3 days with TGZ at concentrations of 0.0.1× C_max_ or 1× C_max_. Cell culture supernatants were collected, and the released alanine aminotransferase (ALT) amount was quantified using the Human ALT SimpleStep ELISA^®^ Kit from Abcam (ab234578) according to the manufacturer’s instructions.

### 2.7 Quantitative PCR

RNA was extracted using the Arcturus^®^ PicoPure^®^ RNA Isolation Kit (Applied Biosystems^TM^ by Life technologies^TM^) according to the manufacturer’s instructions. Reverse transcription was performed using OneScript^®^ RT Mix for qPCR w/gDNAOut (Ozyme), followed by amplification using ONEGreen^®^ FAST qPCR Premix (Ozyme) according to the manufacturer’s instructions. Primers (IL-10, IL-23, IL-12a, TNF-α, TGF-β, and GusB) were purchased from Bio-Rad. Quantitative PCR was performed on QuantStudio 5 Dx (Applied Biosystems by Thermo Fisher Scientific). All CTs were collected, and ∆CT was calculated by subtracting to GusB (housekeeping gene) CT. The relative expression to GusB for each gene was calculated by using the formula RE = 2^−ΔΔCT^.

### 2.8 Graphs and statistics

Plots and statistics were generated using GraphPad Prism v9 (Dotmatics, San Diego, CA) and, otherwise, Excel (Microsoft Office 364).

## 3 Results

### 3.1 The differentiation of monocytes into educated macrophages and dendritic cells is dependent on the age and sex of the subject

**FIGURE 1:**
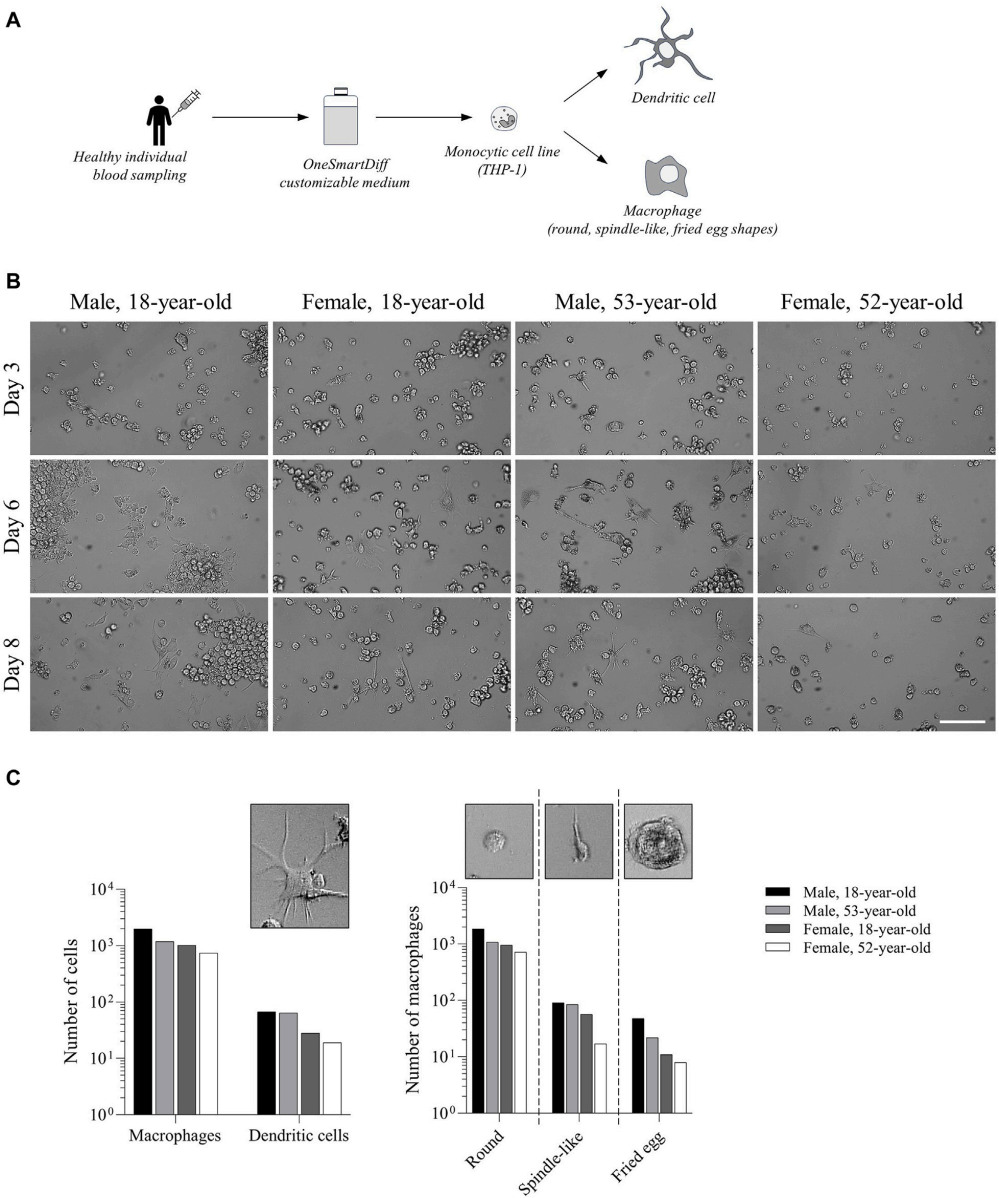
Differentiation of monocytes into educated macrophages and dendritic cells is dependent on the age and sex of the subject. **(A)** Workflow showing THP-1 monocyte differentiation in the customizable OneSmartDiff medium. **(B)** Bright-light microscopy showing the morphology of differentiated monocytes in OneSmartDiff media prepared from an 18-year-old male, an 18-year-old female, a 53-year-old male, and a 52-year-old female. Pictures were taken on days 3, 6, and 8. Scale bar: 100 µm. **(C)** Quantification of the number of monocyte-derived macrophages and dendritic cells (DCs) based on their morphologies and under each culture condition.

In order to predict immune-mediated iDILI, we added educated autologous immune cells into our previously established hepatic spheroid system. These educated immune cells were prepared from the THP-1 cell line that we differentiated with autologous OneSmartDiff media into autologous educated macrophages and DCs (Figure 1A). We demonstrated that the differentiation of monocytes into macrophages and DCs was person-dependent (Figure 1B). Under all conditions, approximately 97% of differentiated monocytes were macrophages, while only 3% were DCs. Interestingly, we observed that the number of monocyte-derived macrophages and DCs was higher in males than that in females, and it was also higher in young adults (18 years) than that in older adults (53/52 years) (Figure 1C). Next, we monitored the morphology of THP-1-derived macrophages under different conditions and found three typical primary morphologies: round, spindle-like, and fried egg shapes (Phuangbubpha et al., 2023). Again, for both types, we observed that their number was higher in males than in females, and it was also higher in young adults than in older adults (Figure 1C). We then analyzed the expression of a panel of cytokines (IL-10, IL-12a, IL-23, TNF-α, and TGF-β) and found that autologous educated immune cells displayed an expression profile that is individual-dependent, suggesting an inter-individual difference in the activation state of these cells (Supplementary Figure S4). Interestingly, we also found that hepatotoxicant agents, such as troglitazone, altered the expression of these cytokines in a person-dependent manner (Supplementary Figure S4). Taken together, our results confirmed that the customizable OneSmartDiff medium could be used to produce autologous educated immune cells that we could add to our educated hepatic spheroid to assess iDILI risk.

### 3.2 Educated spheroids detect iDILI at a therapeutic dose of troglitazone

**FIGURE 2:**
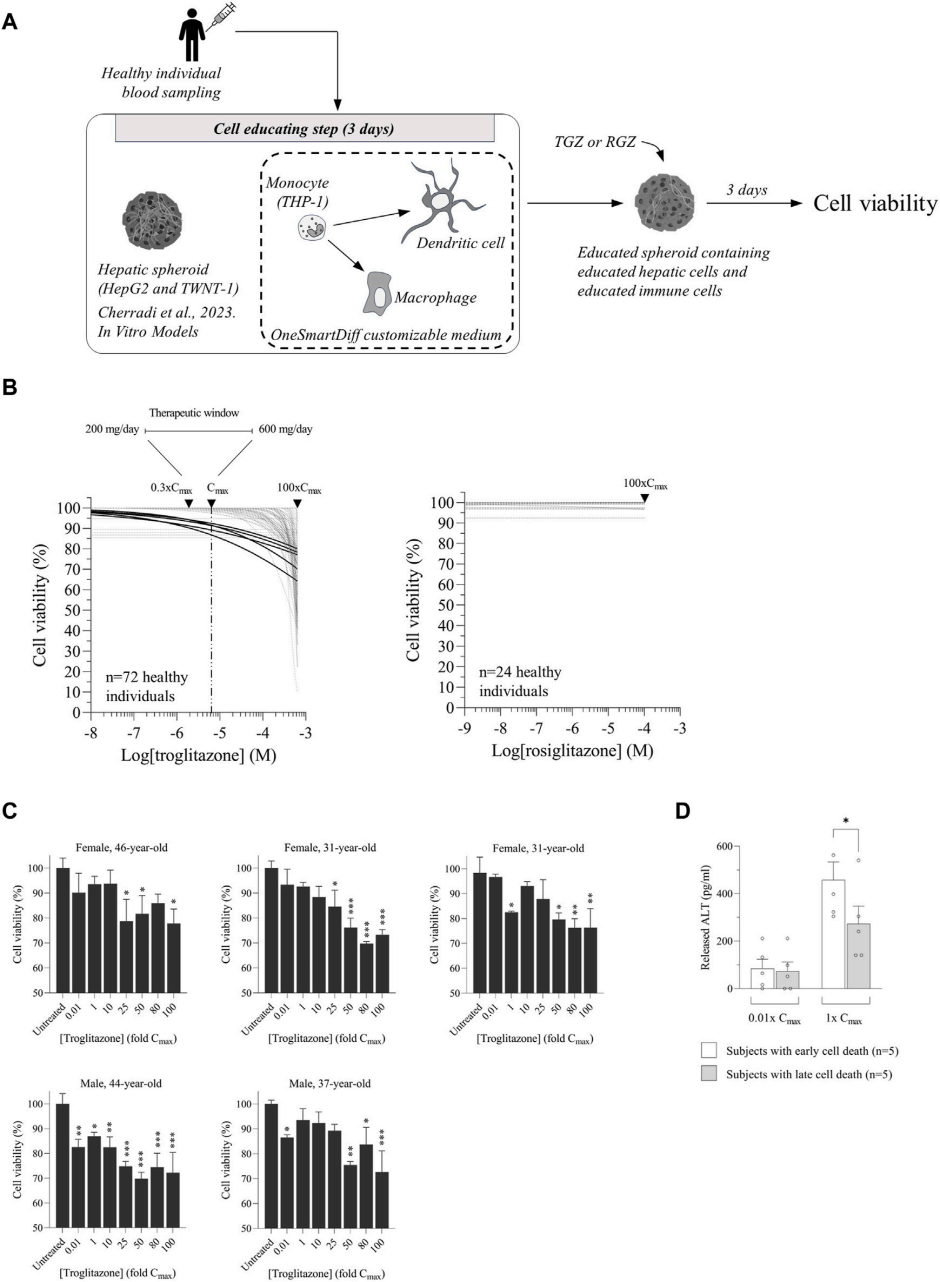
Educated spheroids detect idiosyncratic drug-induced liver injury (iDILI) at therapeutic doses of troglitazone (TGZ). **(A)** Workflow of hepatic spheroids and immune cell educating technology. **(B)** Troglitazone and rosiglitazone were tested on educated spheroids from a cohort of 72 and 24 healthy donors, respectively. Inhibitory curve fits are shown. Only five subjects displayed toxicity with TGZ at concentrations starting from below C_max_. **(C)** Cell viability was reported at different C_max_ values, showing that the toxicity was independent of the doses. **(D)** The released alanine aminotransferase (ALT) level was measured by ELISA after 3 days of treatment with TGZ at concentrations of 0.01× C_max_ and 1× C_max_. Data of five individuals with early cell death and five subjects with late cell death are shown. The results are expressed as pg/mL. ^∗^ *p* < 0.05 Fisher’s *t*-test.

In order to analyze the iDILI risk of troglitazone and its non-iDILI partner compound RGZ, we added educated autologous macrophages and DCs into our educated hepatic spheroids and then treated them with different concentrations of TGZ or RGZ (Figure 2A). Under each condition (i.e., each healthy donor; Supplementary Table S1), cell viability was measured, and an inhibitory dose–response curve fit was generated. As expected, all spheroids generated from healthy donors showed a reduction in cell viability when treated with TGZ, while we did not observe any cell death with RGZ, even with concentrations up to 100× C_max_ (Figure 2B, Supplementary Figure S1). Interestingly, we noted that only five donors showed a decrease in cell viability at TGZ concentrations below C_max_ (Figure 2B). Moreover, we noticed that the cell death caused by TGZ in those five individuals was independent of the doses (Figure 2C), which was in line with the evidence that iDILI is not dose-dependent (Fontana, 2014). Finally, we quantified the amount of released ALT upon TGZ treatment from five subjects who showed early cell death (i.e., below 1× C_max_) and compared it with another five individuals who displayed late cell death (i.e., above 1× C_max_). As expected, we found significantly more ALT released in the early cell death group *versus* the late cell death group, confirming a more pronounced liver cell injury (Figure 2D).

To confirm that our educated spheroid system can detect immune-mediated iDILI, we tested diclofenac, a nonsteroidal anti-inflammatory drug, on a cohort of 24 healthy donors and compared the results to ASA, its non-iDILI partner. ASA is categorized as a clinical compound with low DILI concern (Proctor et al., 2017). Our analysis in a cohort of 24 donors revealed that 1 out of 24, a 19-year-old female, showed cell death only at concentrations above C_max_, demonstrating that ASA could be toxic at higher doses (Supplementary Figure S2). Diclofenac is categorized as a clinical compound with high DILI concern compared to TGZ, which is a clinical molecule with severe DILI concern (Proctor et al., 2017), and is known as an immune-mediated iDILI compound (Yano et al., 2012). Diclofenac has different C_max_ values depending on its galenic formulation and the fed/fasting condition (Chen et al., 2015), with a recommended maximal dose of 150 mg daily. As expected, we found that 20% of subjects (5 out of 24) displayed cell death with concentrations below 100× C_max_, suggesting that diclofenac is a DILI-risk compound (Cherradi et al., 2023). More importantly, we noted that one subject, a 45-year-old male, showed toxicity already at concentrations below C_max_, confirming the iDILI risk of diclofenac (Supplementary Figure S3).

**FIGURE 3:**
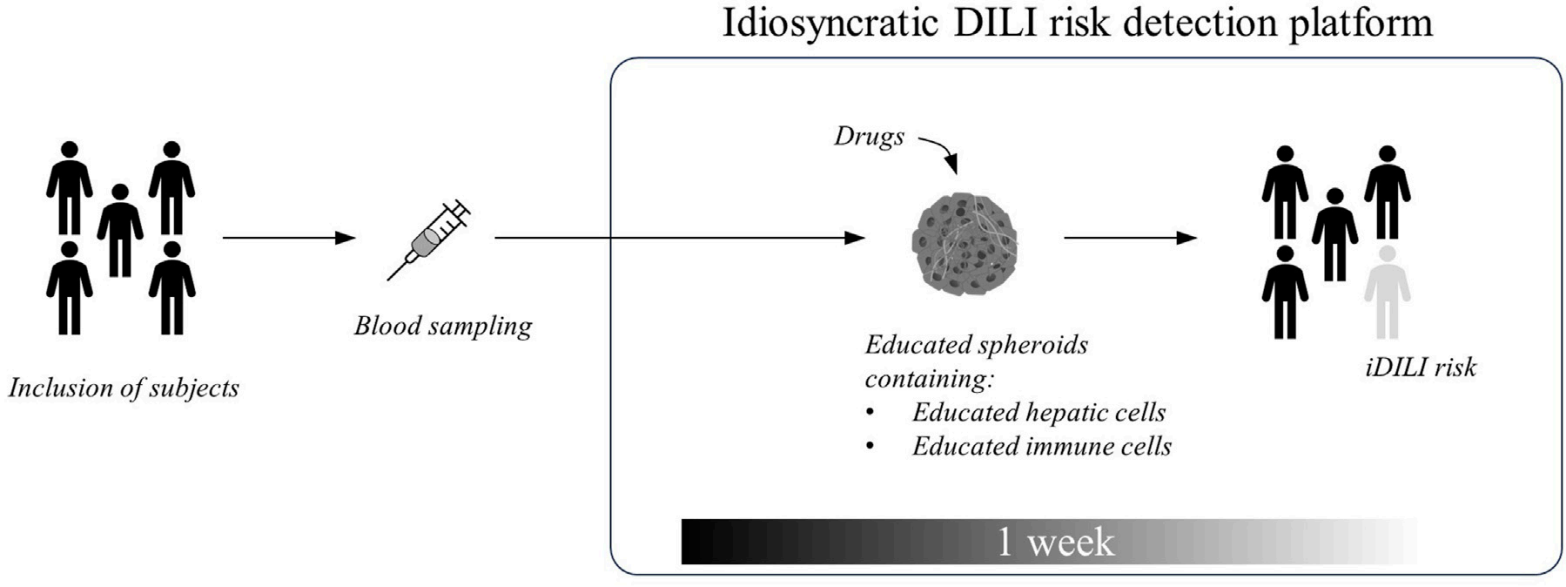
Proposed high-throughput screening of the iDILI risk platform for drug discovery.

Taken together, our results demonstrated that our educated spheroid system can detect the iDILI risk of TGZ in a larger group of subjects, suggesting that this system constitutes a valuable platform for the HTS of the iDILI risk of new therapeutics in cohorts of individuals representative of the population, or it may be used as a companion test to quickly identify patients at risk for iDILI development before initiating a treatment protocol (Figure 3).

## 4 Discussion

Idiosyncratic DILI is a multifactorial event that relies on host factors, including age and sex, and intrinsic biological environments, making it difficult to detect during drug development. In humans, its unpredictable occurrence constitutes a drawback for FDA-approved drug use. Indeed, a large number of drugs were removed from the market because of iDILI, although they were considered safe during their development. Troglitazone belongs to one of these cases. We presented here the first easy-to-handle and quick-to-generate educated spheroid system that is capable of detecting the iDILI risk of TGZ and diclofenac in a large group of people, opening perspectives for a more reliable way to assess iDILI risk at early stages of drug development. Our system also paves the way for a clinically personalized approach to identify patients at risk for iDILI prior to treatments.

Studies that use existing preclinical models to analyze the DILI risk of a drug are generally performed at concentrations that are higher than one magnitude above the clinical concentrations (i.e., up to 100× C_max_) (Proctor et al., 2017). Generally, if cell death occurs before 100× C_max_, a drug is considered a DILI-risk compound. This binary analysis is applicable to conclude on a safety or a risk feature of a molecule, but it cannot address the issue of iDILI. More importantly, these elevated concentrations that are tested *in vitro* are out of the therapeutic window of the drug, making it impossible to predict how safe that molecule is when it is used to treat patients. Indeed, testing a drug at concentrations that are higher than one order of magnitude above clinically relevant concentrations is potentially problematic to predict whether this molecule is safe at therapeutic doses because the mechanisms of toxicity may differ between concentrations at C_max_ and at higher C_max_. ASA, a non-iDILI partner of diclofenac (Granitzny et al., 2017), is believed to be safe. However, it has been shown in a case report of a 41-year-old woman that at a high dose, ASA induced liver injury (Laster and Satoskar, 2014). Our data showed that a 19-year-old female displayed cell death at concentrations above C_max_, confirming that ASA could be toxic at higher doses. In healthy volunteers, troglitazone, given per os at therapeutic doses of 200–600 mg/day, reaches peak plasma drug concentrations of 0.9–2.8 mg/L approximately 2–3 h later (Plosker and Faulds, 1999), corresponding to 0.3× C_max_ and 1× C_max_, respectively. Our data clearly demonstrated that TGZ induced an iDILI risk in 5 subjects out of a cohort of 72 within the therapeutic window. Unfortunately, we could not examine whether the five individuals that were identified with iDILI risk have true iDILI susceptibility as the study was conducted on healthy individuals and as TGZ was withdrawn by the FDA from the market in 2000. Nevertheless, a retrospective study showed that TGZ caused liver failure in 94 out of the 1.92 million treated patients, and less than 5% of them developed liver injury with elevated ALT levels (Graham et al., 2003). Our analysis of TGZ-mediated iDILI in a cohort of 72 healthy donors revealed that only 5 subjects out of the 72 (6.9%) showed toxicity at concentrations below C_max,_ confirming the proportion of iDILI detected in clinical trials. Interestingly, the US Government Accountability Office (GAO) published a letter on 19 January 2001 reporting that among the drugs that were withdrawn, TGZ had a greater liver injury risk for women. Our data showed that among the five patients with iDILI, three were females and two were males, confirming that females are at a higher risk for iDILI than males. To the best of our knowledge, the educated spheroid model is a unique preclinical model that is capable of detecting cell toxicity at therapeutic doses, suggesting that this system could be used as a companion test to identify a patient who could be at risk for iDILI occurrence. Our data encourage us to initiate a prospective study on patients treated with drugs other than TGZ, as TGZ was withdrawn in 2000, to confirm the accuracy of this personalized model with the aim of its use in a personalized approach for iDILI risk.

Presently, studies on immune cell functions were conducted with differentiated macrophages and DCs that were prepared under non-physiological conditions (Villar et al., 2023). This approach is not relevant as the differentiation of these cells in humans depends on host factors and environments that are not reproduced *in vitro* (Kenna and Uetrecht, 2018). The OneSmartDiff customizable medium offers the possibility to simultaneously differentiate monocytes into macrophages and DCs mimicking the immune cell populations that are present in clinical samples. Kawase et al. previously reported that NSAID-induced liver toxicity is dependent on the differentiation status of macrophages (Kawase et al., 2022). The OneSmartDiff medium could be prepared for each individual; it is, therefore, possible to generate simultaneously educated immune cell populations and educated hepatic cells to reproduce the biological conditions for each subject. Our results show that the number of monocyte-derived macrophages and DCs was higher in males than that in females, and it was also higher in young adults (18 years) than in older adults (53/52 years old), which are in line with previous works, where it was reported that the number of macrophages is significantly lower in adults than in children and young adults (Ogawa et al., 2000), as well as in females than in males (Chen et al., 2021). Moreover, our data confirmed that geriatric conditions brought from the customizable OneSmartDiff media that have been prepared from depleted sera from older adults somehow prevent macrophage differentiation (Mahbub et al., 2012). Finally, our data revealed that educated immune cells may trigger an inter-individual difference in the cytokinome landscape that could modulate iDILI risk occurrence. Further studies are needed to explore the link between the cytokine profile and iDILI risk. All together, we believe that the integration of all biological information from the same person within a spheroid makes it possible to assess iDILI risk in a cohort of subjects. The same strategy could also be used to generate macrophages and DCs under pathological conditions to analyze their functions in a manner that is closer to what occurs in patients.

It is well known that some host factors, including age and sex, could increase the risk of iDILI occurrence in a drug-specific way (Yamashita et al., 2017). Unfortunately, these parameters are, so far, under-considered in preclinical models. Attempts were made using animal models. However, data were not satisfactory to reliably anticipate iDILI risk in humans (Atkins et al., 2020). Our data clearly demonstrated that our system of educated spheroids, which is person-dependent, could address the issue and offers a way to analyze the iDILI risk in individuals for whom a medication is dedicated.

Primary human hepatocytes are considered a gold standard for DILI analysis. However, their limited availability, the requirement of invasive procedures to collect, and their quick dedifferentiation when cultured *in vitro* make them unsuitable for HTS. Alternatives were proposed with human hepatoma cell lines, such as HepG2 or HepaRG. HepaRG cells are closer to PHHs as they express greater levels of phase I and phase II drug-metabolizing enzymes than HepG2. Nevertheless, it has been reported that HepG2 spheroids could identify 58% of DILI-positive molecules, while HepaRG spheroids could identify only 47% of these compounds on a panel of 150 DILI drugs (Basharat et al., 2020). We are aware that the expression of drug-metabolizing enzymes is lower in HepG2 cells than in PHHs. Nevertheless, we previously published data showing that educated HepG2 spheroids have increased basal CYP activity, which was further enhanced upon exposure to drugs (Cherradi et al., 2023). Moreover, transcriptomic profiling of educated spheroids revealed that the expression of some phase I and phase II drug-metabolizing enzymes is comparable to the liver tissue (unpublished data). Our data suggest that HepG2 cells could be used as alternative cells to PHHs to assess iDILI in our system. The model could be strengthened further by adding endothelial cells as these cells are potential targets of DILI, and a larger panel of iDILI and non-iDILI drugs should be tested with the system. Experimentally, this is a massive amount of work as each drug must be tested on cohorts with a large sample size. Finally, further analysis of the metabolism, for instance, by measuring the induction of drug-metabolizing enzymes and their activities, should be done to better understand the mechanism of iDILI of these compounds.

In summary, we present here an educated spheroid system containing autologous educated macrophages and DCs that can detect the iDILI risk of TGZ and diclofenac at therapeutic concentrations. As the system does not require primary cells and is easy and quick to set up, it could be used for HTS of new molecules supporting drug-development pipelines of pharmaceutical companies.

## Data Availability

The original contributions presented in the study are included in the article/Supplementary Material; further inquiries can be directed to the corresponding author.
