# Supplementary figures and images for: Exploiting the predictive power of educated spheroids to detect immune-mediated idiosyncratic drug-induced liver injury: the case of troglitazone
Source: Front Pharmacol. 2024 Apr 10;15:1378371. doi: 10.3389/fphar.2024.1378371 (PMC11039894; doi:10.3389/fphar.2024.1378371)

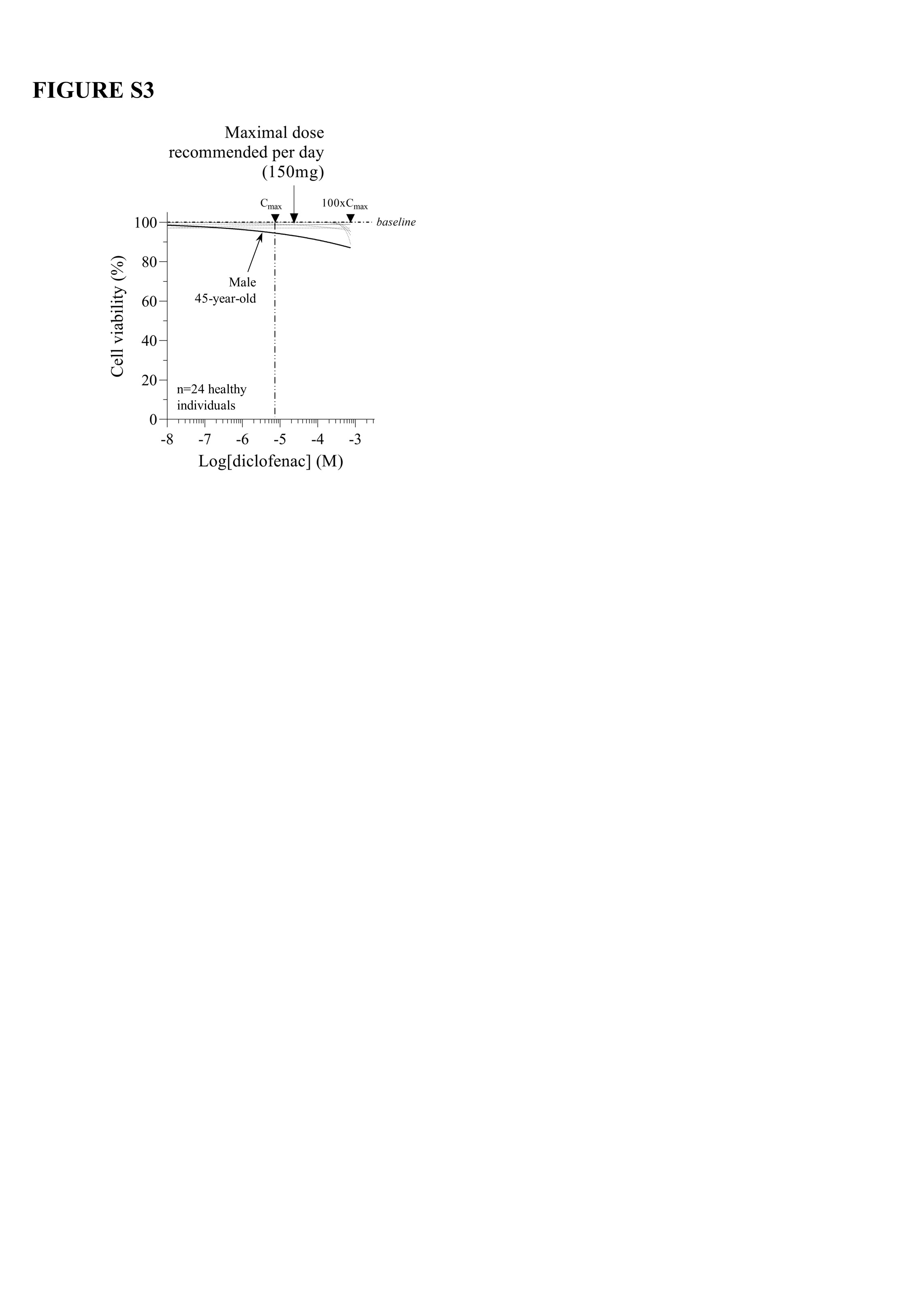

Supplement: Supplementary file 1 [file Image3.JPEG]

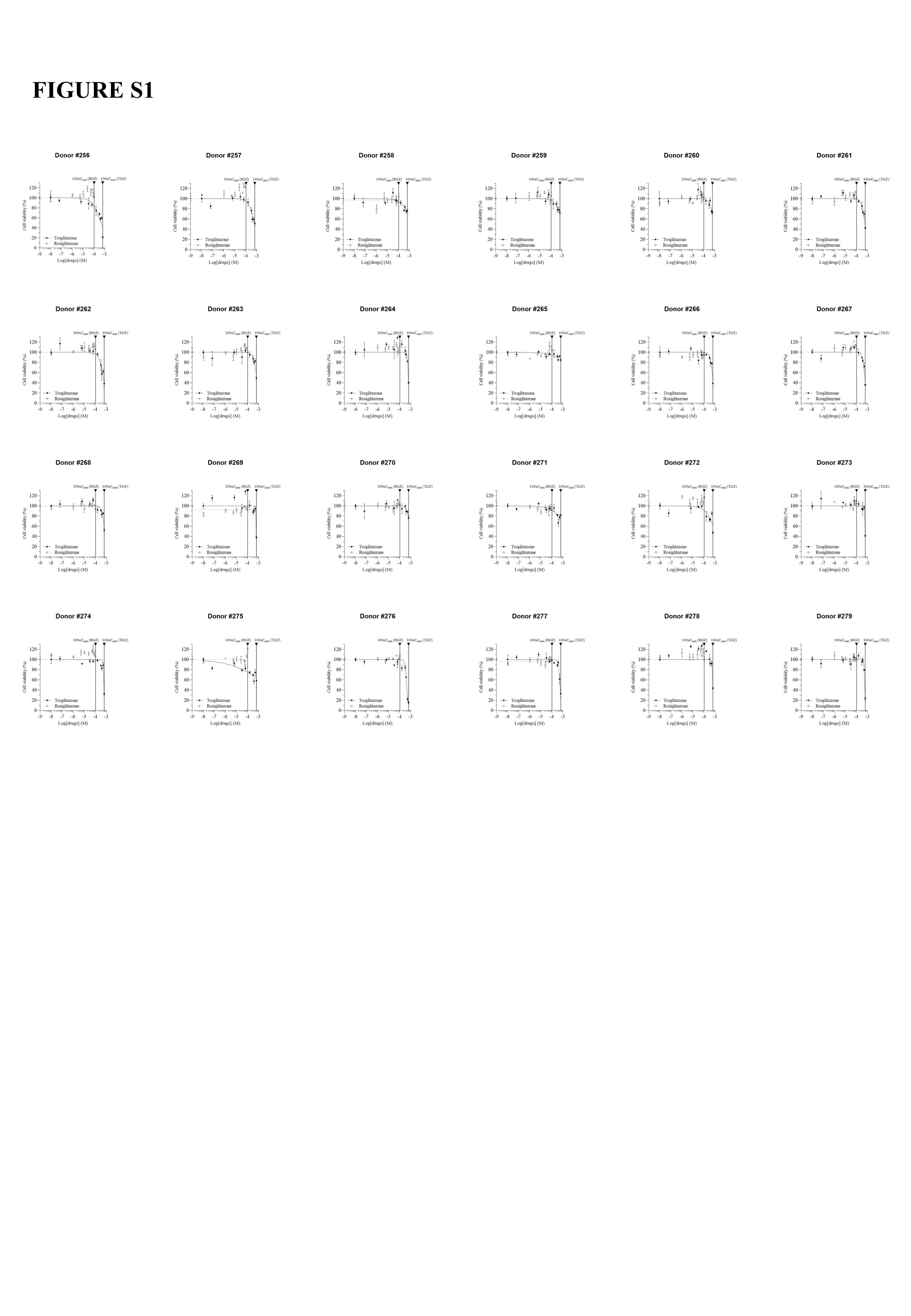

Supplement: Supplementary file 2 [file Image1.JPEG]

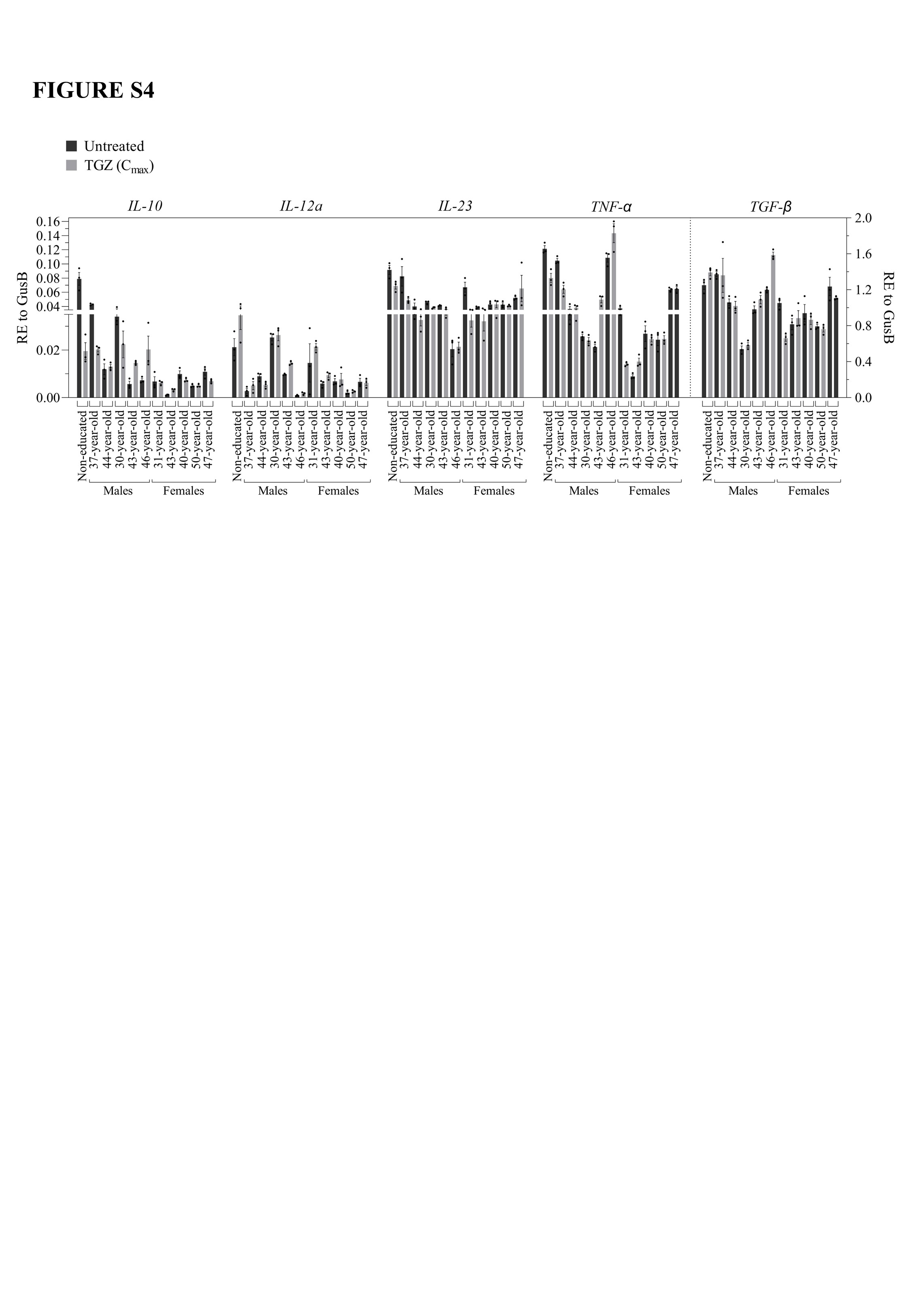

Supplement: Supplementary file 3 [file Image4.JPEG]

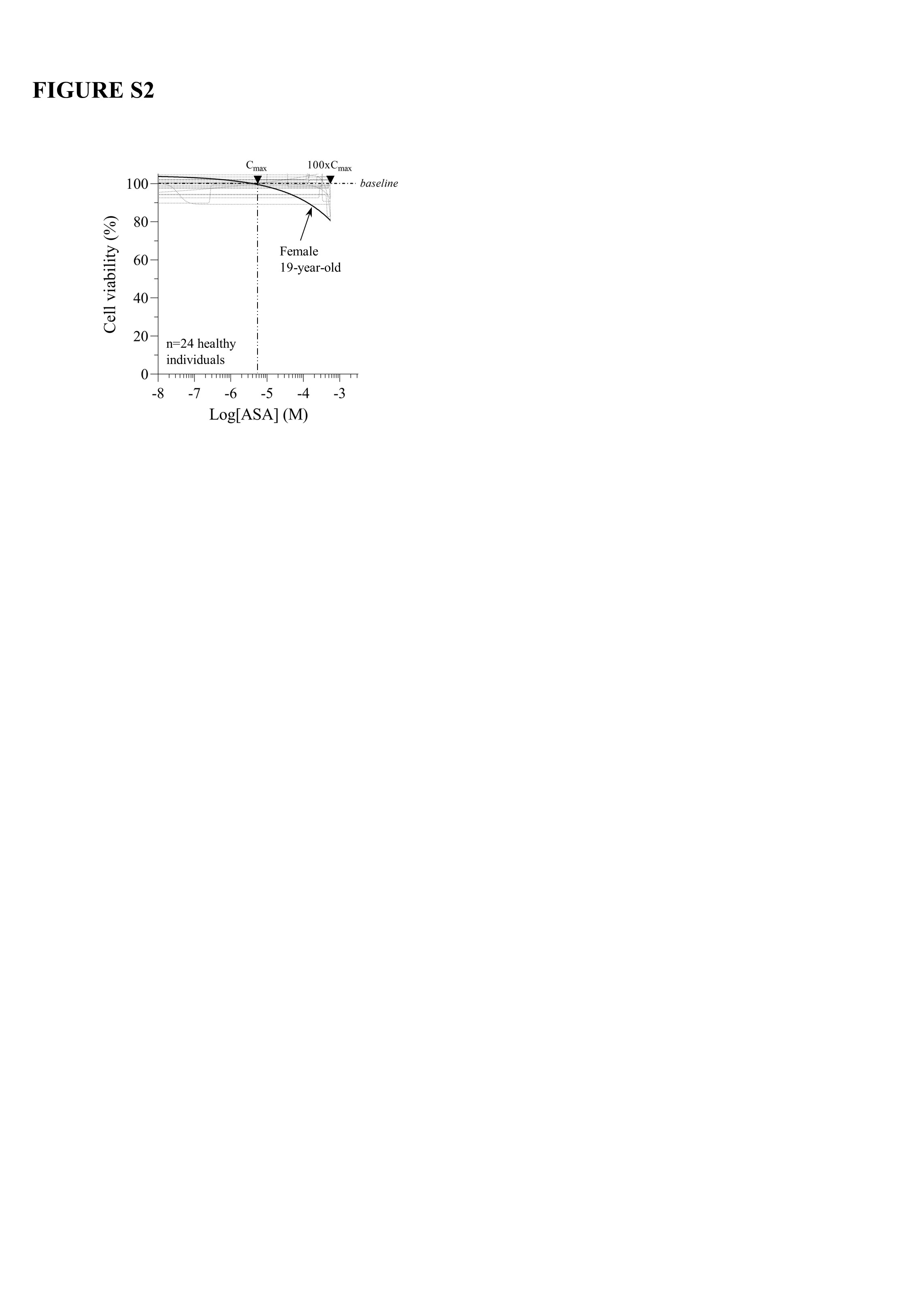

Supplement: Supplementary file 4 [file Image2.JPEG]
